# Comparative analysis of asymptomatic infection prevalence in Beta, Delta, and Omicron surges of COVID-19

**DOI:** 10.1016/j.bjid.2024.103724

**Published:** 2024-02-13

**Authors:** Mohammad Jafari, Ahmad Jabrodini, Aliyar Pirouzi, Ahmad Meshkin, Mehdi Mohsenzadeh

**Affiliations:** a Cellular and Molecular Research Center, Gerash University of Medical Sciences, Gerash, Iran; b Education Development Center, Committee of Medical Education Development, Gerash University of Medical Sciences, Gerash, Iran

**Keywords:** SARS-CoV-2, Asymptomatic individuals, Beta surge, Delta surge, Omicron surge

## Abstract

**Background:**

The COVID-19 pandemic, caused by the SARS-CoV-2 virus, has had a devastating impact on the global population, with an estimated 650 million people infected and more than 6.6 million lives lost. Asymptomatic individuals have been shown to play a significant role in the transmission of the virus. Therefore, this study aims to investigate and compare the prevalence of asymptomatic individuals across three waves associated with the Beta, Delta, and Omicron variants of the virus.

**Methods:**

This retrospective study was conducted between December 2020 and March 2022. The study population consisted of passengers on international flights who were referred to the Gerash Clinical and Molecular Diagnosis Laboratory. Real-time PCR was employed for the diagnosis of SARS-CoV-2.

**Results:**

Out of a total of 8592 foreign travelers referred to our laboratory, 139 (1.16 %) tested positive for SARS-CoV-2 infection and were asymptomatic. During the Beta surge, 35 (1.49 %) out of 2335 passengers tested positive for SARS-CoV-2. In the Delta surge, 31 (0.6 %) out of 5127 passengers tested positive. However, during the Omicron surge, a significantly higher number of passengers, specifically 73 (6.46 %) out of 1130, had a positive result for the SARS-CoV-2 test.

**Conclusion:**

Considering the significant role of asymptomatic transmission in the spread of COVID-19, it is imperative to reconsider health policies when dealing with future surges of the Omicron subvariants. Additionally, we strongly recommend that the World Health Organization prioritize the development and distribution of second-generation vaccines that target not only disease but also infection prevention.

## Introduction

COVID-19 (Coronavirus Disease 2019) is caused by the Severe Acute Respiratory Syndrome Coronavirus 2 (SARS-CoV-2) strain, which the WHO declared a global pandemic on March 11, 2020.^1^ The strain B.1.1.529 (Omicron) is one of the SARS-CoV-2 strains that was first reported in Botswana and South Africa.^2^ Previous studies showed that Omicron evades binding and neutralization by most therapeutic SARS-CoV-2 monoclonal antibodies and also neutralizing antibodies induced by vaccination or prior infection.^3^^,^^4^ Other research has demonstrated that mutations within the spike protein of the Omicron variant have increased transmissibility even among some vaccinated people.^4^ More than thirty mutations have been identified in the spike gene of SARS-CoV-2. These mutations allow the virus to increase transmission rate, disease severity, and immune evasion abilities.^5^ Other factors that cause the rapid transmission of Omicron variant include low vaccination coverage, changes in social behavior, and lack of or improper use of masks.^6^

The early widespread dissemination of Omicron shows the urgency of better understanding the transmission dynamics of this variant. The transmission of the virus by infected individuals who are asymptomatic has been one of the controversial topics discussed Since the outbreak of COVID-19.^7^ This issue has also been argued in relation to Beta and Delta strains, but due to the many mutations occurring in the spike gene of Omicron strain and the fact that this part of the virus plays important role in binding and entry of the virus in target cells, so studying the spread of this virus by asymptomatic people can be used to improve public health policies. Therefore, the aim of the current study is to compare the frequency of asymptomatic infections in passengers on foreign flights with triple waves caused by Beta, Delta, and Omicron strains.

## Method and material

This retrospective study was conducted from December 2020 to March 2022. The study included all people who, as passengers of foreign flights, applied for a corona diagnostic test at the Gerash clinical and Molecular Diagnosis Laboratory. All participants were asymptomatic, and if they exhibited any symptoms of coronavirus infection, they were not sampled at our facility. Instead, they were referred to a clinic where sampling for suspected COVID-19 cases was performed.

After collecting demographic data, samples were collected from the throat and nasal mucosa using sterile swabs. Samples were transferred to screw-cap tubes containing 2‒3 mL VTM and stored at 4 °C until genome extraction.

The passengers in this study were divided into three groups based on referring time. The first category includes passengers who come for the Corona test from January 10, 2021, to June 6, 2021. This interval coincided with the fourth wave of COVID-19 disease, which was dominated by the Beta strain. The second category was passengers who referred from June 7, 2021 to January 5, 2022, during the Delta strain outbreak. Passengers who visited during the Omicron strain outbreak between January 6, 2022 and March 23, 2022 were also classified in the third category. It should be noted that in all three batches mentioned above, confirmation of the dominant strain was performed by sending samples to the health reference laboratory of Shiraz University of Medical Sciences.

### Real-time PCR test

The RNA extraction kit from ROJE Technology was used according to the manufacturer's instructions. Real-Time PCR was performed using an Iranian Jetroo kit. In the real-time PCR test, the expression of two viral genes, RdRp and E, were simultaneously evaluated and Human ribonuclease P gene was used as an internal control. Positive results based on Cycle threshold (Ct) less than 35 for RdRP genes and E gene are considered. The test was repeated on a new sample If the E gene alone was positive.

### Statistical analysis

All statistical analyses were performed using Graphpad Prism software (version 6). Frequency of asymptomatic infection and mean comparison of Ct were evaluated using Chi-Squared and Student *t*-test, respectively; *p*-values ≤0.05 were considered to be statistically significant.

## Result

**Fig. 1:**
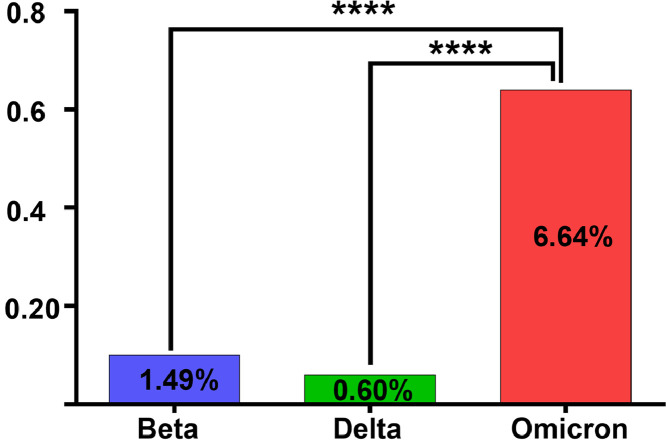
Prevalence of asymptomatic SARS-CoV-2 infection in three consecutive surges.

Our study involved 8592 Iranian passengers, with 6272 men (73 %) and 2320 women (27 %), and average age 37 ± 27 years. Among them, 139 (1.16 %) tested positive. All of these individuals did not exhibit any clinical symptoms at the time of sampling, and even after 24 h when they visited the laboratory to receive their results, they remained symptom-free. Individuals who subsequently developed clinical symptoms were excluded from the comparison among the groups. Among the 8592 passengers, 2335 people belonged to the first category (Beta group), of which 35 (1.49 %) people had positive tests for Corona. The second group (Delta group) contained 5127 people, of whom 31 (0.6 %) people tested positive. The third group (Omicron group) included 1130 individuals, of which 73 individuals (6.46 %) were declared positive (Table 1). A significant difference in the frequency of infections was observed between the three groups (*p*-value < 0.00001). In a pairwise comparison between the three groups, the differences were significant [between the Beta group and the Omicron *p*-value < 0.00001 (OR = 4.72, 95 % CI 3.14‒7.1) and between the Delta group and the Omicron *p*-value < 0.00001 (OR = 11.4, 95 %CI 7.74‒18)] (Fig. 1).

**Table 1:** Time of triple Beta, Delta and Omicron waves and frequency of asymptomatic people in triple waves.

Surges	Start time of Surge	End time of Surge	Performed Test	Positive Test	Percentage
Beta	10 Jan 2021	6 Jun 2021	2335	35	1.49 %
Delta	7 Jun 2021	5 Jan 2022	5127	31	0.6 %
Omicron	6 Jan 2022	23 Mar 2022	1130	73	6.46 %
Total			8592	139	1.61 %

The average Ct values of RdRp gene in Beta, Delta, and Omicron surge was 30.29, 25.98, and 27.40, respectively. Comparing the average of the three groups studied showed that the Ct value in the triple waves had a significant difference. So that in the wave caused by the Beta strain, the mean of Ct was significantly lower than the other two waves.

## Discussion

The frequency of asymptomatic people during three successive Beta, Delta, and Omicron waves in Gerash city was examined in this study. A comparison of the SARS-CoV-2 infection frequency in passengers on foreign flights in all three waves showed that asymptomatic people increased more than 4 times in the Omicron wave when compared to the Beta wave and increased more than 10 times compared to the Delta wave. According to studies done before the Omicron outbreak, 30–40 % of SARS-CoV-2 infections are asymptomatic.^8^^,^^9^ As vaccination coverage expanded, particularly after a booster dose, the incidence of severe symptoms decreased in the vaccinated population, while Omicron infection occurs in a higher percentage of people infected with no clinical symptoms or with mild symptoms.^10^^,^^11^ The notable changes in the protein S mutations of the Omicron subvariants, particularly within the N-terminal region and receptor-binding domain, could be attributed to the observed changes. These specific regions are known to harbor crucial neutralizing antibody epitopes.^12^ These mutations have been found to confer increased resistance to neutralizing antibodies and are linked to immune evasion.^13^ On the other hand, mutations in the Omicron RBD have led to a 2.5-fold stronger binding to ACE2 compared to prototype SARS-CoV-2,^14^ indicating an adaptive evolution of SARS-CoV-2 in humans to enhance viral transmission.^8^ Furthermore, some other studies have shown that compared to previous strains of SARS-CoV-2, the Omicron strain has a shorter incubation period and similar or milder clinical symptoms,^15^ while having about 10 times higher infectivity.^16^ One of the most important factors that increase the infectivity of viruses is the transmission of infection from asymptomatic populations.^7^ Studies examining the number of asymptomatic people infected with the SARS-CoV-2 virus in different communities have shown various results depending on the design and method used, age, geographic region, and vaccination status of the study subjects.^17^ Furthermore, the percentage of these individuals was reported to vary in successive waves caused by different strains of SARS-CoV-2.^7^

Real-Time PCR is now a commonly used and accepted method in the majority of country to detect SARS-CoV-2 infection.^18^ Real-time PCR assays use a cycle threshold Ct indicator to estimate viral load, thus, a lower Ct indicates a higher viral load. Although some studies have claimed that the interpretation of viral load by Ct values is incorrect,^19^ most studies have used the Ct value to assess viral load. This study and Jefferson et al. showed that many asymptomatic carriers had high viral titers in the throat and nose based on relatively low Ct values in real-time PCR assays.^9^ In this study, the mean Ct of individuals identified in the Omicron wave was significantly lower than the Beta wave, while there was no significant difference from the average Ct of individuals identified in the Delta wave.

In general, the ability to detect viral infection at the time of sampling is one advantage of the Real-Time PCR method compared serology tests. Therefore, it shows the frequency of people infected with the virus in a more accurate way. Because it is impossible to distinguish between the duration of virus exposure and the immunity brought on by the vaccination injection when measuring the antibody level. On the other hand, due to the weak virulence of Omicron strain and patients who are mostly asymptomatic or have mild disease, Real-Time PCR to detect infection with this strain is more suitable than chest X-Ray imaging or computed tomography scan of the chest.

## Conclusion

Since asymptomatic people are the main route of transmission of the COVID-19 disease, it therefore seems necessary to revise health policies in dealing with Omicron substrain in future waves of the disease. On the other hand, the high frequency of asymptomatic people in the Omicron wave suggests that second-generation vaccines should be a priority for the World Health Organization to prevent infection, not just disease. Enhanced surveillance with rapid screening tests and full sequencing of viral genome should also be considered.

## Funding

This study was carried out with the financial support of Gerash University of Medical Sciences.

## Ethical approval

This study received ethical approval from the Ethics Committee of Gerash University of Medical Sciences under the code IR.GERUMS.REC.1402.012. Before analysis, patient laboratory data underwent anonymization and de-identification to ensure confidentiality and privacy.

## Declaration of generative AI and AI-assisted technologies in the writing process

During the preparation of this work, the authors used Chat GPT in order to improve language. After using this tool, the authors reviewed and edited the content as needed and takes full responsibility for the content of the publication.

## Conflicts of interest

The authors declare no conflicts of interest.
